# Stability of non-metal dopants to tune the photo-absorption of TiO_2_ at realistic temperatures and oxygen partial pressures: A hybrid DFT study

**DOI:** 10.1038/s41598-019-47710-7

**Published:** 2019-08-06

**Authors:** Pooja Basera, Shikha Saini, Ekta Arora, Arunima Singh, Manish Kumar, Saswata Bhattacharya

**Affiliations:** Department of Physics, Indian Institute of Technology Delhi, New Delhi, 110016 India

**Keywords:** Photocatalysis, Photocatalysis, Electronic structure, Electronic structure, Electronic structure

## Abstract

TiO_2_ anatase is considered to play a significant importance in energy and environmental research. However, for developing artificial photosynthesis with TiO_2_, the major drawback is its large bandgap of 3.2 eV. Several non-metals have been used experimentally for extending the TiO_2_ photo-absorption to the visible region of the spectrum. It’s therefore of paramount importance to provide theoretical guidance to experiment about the kind of defects that are thermodynamically stable at a realistic condition (e.g. Temperature (*T*), oxygen partial pressure (${{\boldsymbol{p}}}_{{{\bf{O}}}_{{\bf{2}}}}$), doping). However, disentangling the relative stability of different types of defects (viz. substitution, interstitial, etc.) as a function of charge state and realistic *T*, ${{\boldsymbol{p}}}_{{{\bf{O}}}_{{\bf{2}}}}$ is quite challenging. We report here using state-of-the-art first-principles based methodologies, the stability and meta-stability of different non-metal dopants X (X = N, C, S, Se) at various charge states and realistic conditions. The ground state electronic structure is very accurately calculated via density functional theory with hybrid functionals, whereas the finite *T* and ${{\boldsymbol{p}}}_{{{\bf{O}}}_{{\bf{2}}}}$ effects are captured by *ab initio* atomistic thermodynamics under harmonic approximations. On comparing the defect formation energies at a given *T* and ${{\boldsymbol{p}}}_{{{\bf{O}}}_{{\bf{2}}}}$ (relevant to the experiment), we have found that Se interstitial defect (with two hole trapped) is energetically most favored in the p-type region, whereas N substitution (with one electron trapped) is the most abundant defect in the n-type region to provide visible region photo-absorption in TiO_2_. Our finding validates that the most stable defects in X doped TiO_2_ are not the neutral defects but the charged defects. The extra stability of ${({\bf{S}}{\bf{e}}{\bf{O}})}_{{\bf{O}}}^{+{\bf{2}}}$ is carefully analyzed by comparing the individual effect of bond-making/breaking and the charge carrier trapping energies.

## Introduction

TiO_2_ has attracted worldwide research sensation in the past few decades because of its unique properties and potential applications as a photo-catalyst^1–6^. Anatase is the technologically most relevant polymorph of TiO_2_^7–10^. However, owing to its large bandgap (~3.2 eV), it absorbs only ultra-violet light (*λ* < 400 nm) and shows almost no response to visible and near infra-red light (i.e. 400 ≤ *λ* ≤ 1400 nm). Hence, reducing the band gap of TiO_2_ to coincide with the visible spectrum is an active area of research.

Doping-mediated modulation of the band gap is one of the most pragmatic approaches adopted in the pursuit to improve photo-absorption of TiO_2_ in the visible region^11^. Various dopants viz. transition metals^12–15^, noble metals^16,17^, charge-compensated and uncompensated co-doped elements^18,19^ have been reported in the past. However, these metal dopants often act as recombination centre due to their capacity to induce trapping levels in the band gap and reduce a significant amount of photo-excited carriers^9^.

To avoid such additional trapping levels inside the band gap region, non-metal doping is suggested as an alternative strategy to improve visible-light absorption^5,20–26^. Various non-metal dopants viz. N^27–30^, C^31,32^, S^26,33,34^, Se^35,36^ have already been experimentally reported. However, despite significant amount of research are done both experimentally and theoretically on this system, there exists a lot of controversy relating to most preferred defect site^31,32^. Moreover, it’s still an open question concerning the stability of various types of defects as a function of charge state at realistic conditions (e.g. Temperature (*T*), oxygen partial pressure (${p}_{{{\rm{O}}}_{2}}$) and doping (doping is considered as means of fixing the chemical potential of the electrons (*μ*_*e*_))). Note that the formation energy of one isolated defect can be reduced significantly by several eVs, when the charge carriers (holes or electrons) are available in the material. Moreover, charge-carrier doping (either accidental or intentional) can strongly influence the material’s surface properties^37^. Thus, disentangling the relative stability of different types of charged defects in enhancing photo-catalytic action is quite challenging. It’s therefore of profound importance to provide theoretical guidance to experiment to address the stability of charges. However, until date, theoretical calculations are limited to neutral defects only in TiO_2_ and do not address clearly the case of charged defects.

**Figure 1:**
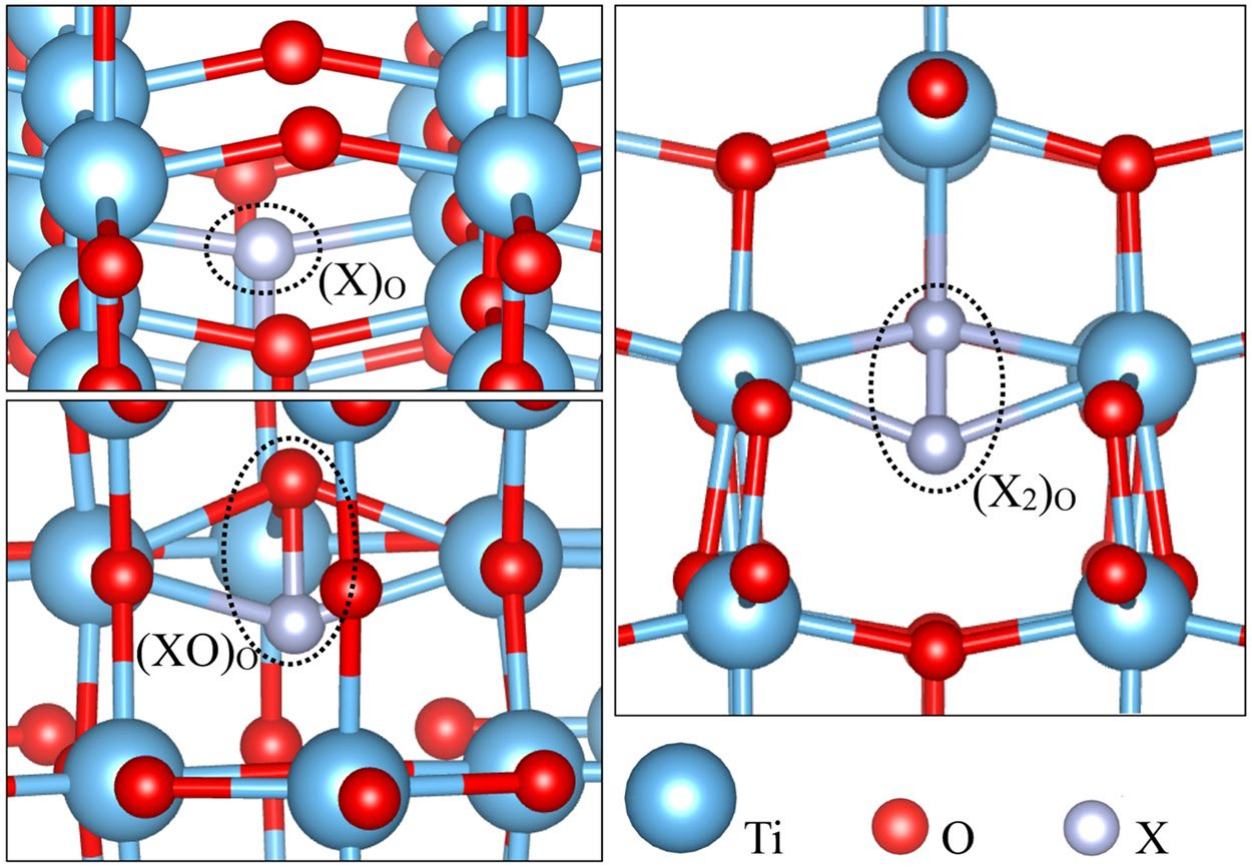
Possible positions of non-metals X (=N, C, S, Se) dopants in anatase TiO_2_ viz. (X)_O_, (XO)_O_ and (X_2_)_O_ (see text for details).

This motivates us to revisit the bulk anatase form of TiO_2_ to elucidate the role of non-metal dopants as a function of charge at a realistic condition. We have used state-of-the-art density functional theory (DFT) with hybrid functionals combined with *ab initio* atomistic thermodynamics approach^38,39^. We have modeled non-metal (X) doped TiO_2_, assuming various types of defect possibilities: (i) the non-metal X can replace O atom making a substitutional defect (X)_O_, (ii) the non-metal X can form interstitial defects (XO)_O_ defect, and (iii) combination of both (i) and (ii) i.e. one X is replacing one O forming substitutional defect, while at the same time one additional X is present as an interstitial at the same site. The latter is denoted as (X_2_)_O_ defect (see Fig. 1). The experimental evidences of the stability and existence of some of the configurations (X)_O_, (XO)_O_, (X_2_)_O_ are confirmed via XPS studies^34,40–44^. It is also noted that, we have considered the case, where we have taken substitution at O-site only. However, S and Se non-metal dopants also have a possibility to substitute at Ti-site. The substitution at Ti site is already studied well^45^, due to which we have limited our study to O-site only. In this article we, therefore, provide an exhaustive study on the relative stability of different types of charged X-related defects (*vide infra*, [(X)_O_]^*q*^, [(XO)_O_]^*q*^ and [(X_2_)_O_]^*q*^ with X = N, C, S, Se; *q* = −2, −1, 0, +1, +2, etc.) in TiO_2_ anatase at various realistic conditions. However, this theoretical work aims on the thermodynamic stability of dopants under realistic temperatures and oxygen partial pressures, while the experimental results sometimes rely on kinetic stability under practical growth conditions, such as the heating rate, holding time and experimental atmosphere. The latter study is beyond the scope of the present paper. Nevertheless, providing a phase diagram by accurate estimation of free energy of formation is very useful for the experimentalists in many instances. Seeing such phase diagram one can get a qualitative information regarding the possible relevant defect states of that concerned material at a given temperature and pressure even if the stability might get changed over time by considering the kinetic effect. Further, we have also given a quantitative analysis to address the underlying reason for extra stability of a specific defect at a given environmental condition by comparing the contribution of bond-making/breaking and charge carrier trapping energies.

## Results and Discussion

### Most stable defect configurations

We have determined the energetically most favorable geometric configurations (X)_O_, (XO)_O_ and (X_2_)_O_ defects optimized at various charge states $q=-\,2,-\,1,0,+\,1,+\,2$ for various non-metal dopants (X = N, C, S, Se) in the Ti_16_O_32_ framework. In our supercell of Ti_16_O_32_ the geometric configurations XTi_16_O_31_□_1_, XTi_16_O_32_ and X_2_Ti_16_O_31_□_1_ represent respectively (X)_O_, (XO)_O_ and (X_2_)_O_. For this purpose, we have adopted an iterative strategy^46^: starting with the Ti_16_O_32_ supercell, we first identified the most favorable sites for the O-□ by scanning over all possible sites. We find that, since they belong to the same symmetry point, all the atomic sites are equivalent. Hence, we can substitute non-metal dopants at any O-site in the system. For interstitial and complex interstitial configurations, we simply consider one oxygen atom in the Ti_16_O_32_ framework and scan its all surrounding positions. The configuration having minimum energy is taken as a final structure for studying defects. Note that for all geometry relaxations we have used PBE functional as the difference in geometry optimized by PBE and HSE06 are insignificant. Therefore, on top of the final relaxed geometry, we have performed HSE06 calculations to determine the correct energetics associated with those optimized structures.

### Validation of DFT functionals

**Figure 2:**
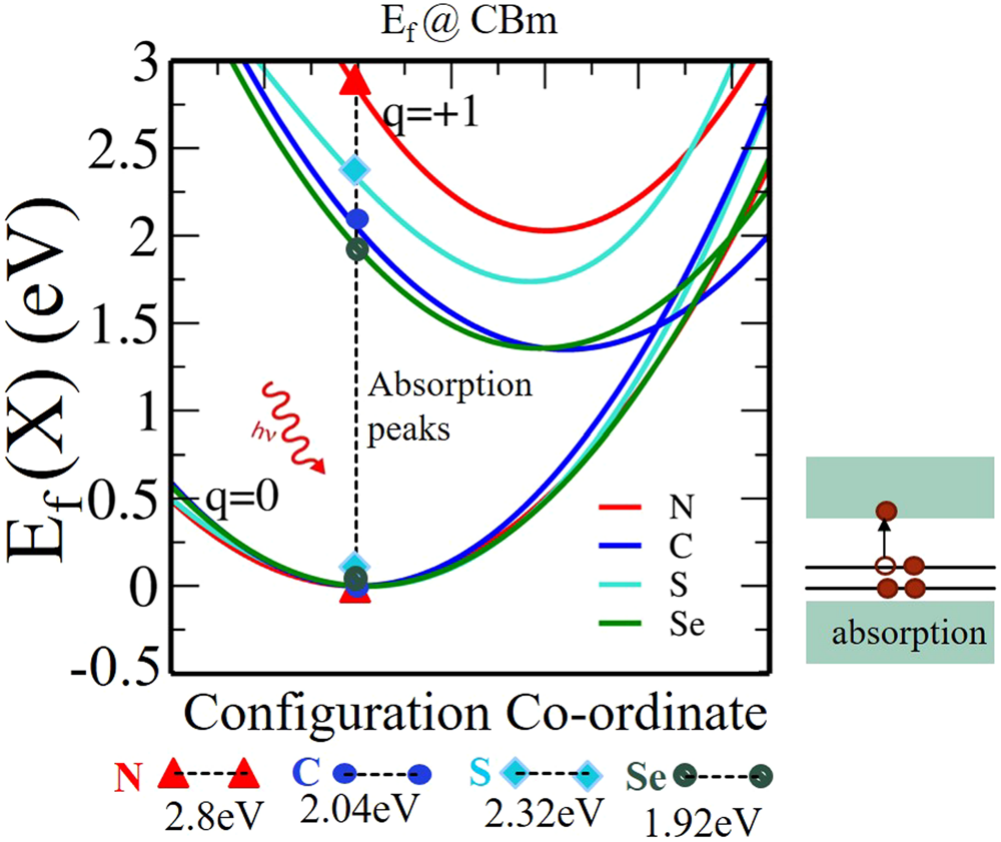
Configuration coordinate diagram for (X)_O_ in Ti_16_O_32_: The HSE06 formation energies in the two different charge states (0, +1) are plotted as a function of the displacement of atoms. The chemical potential of electron *μ*_e_ is taken along the conduction band minimum (CBm).

To ensure that our findings are not an artifact of the chosen treatment of the xc-functional, we have first thoroughly benchmarked the xc-functionals. Note that using PBE, the band gap of TiO_2_ is quite underestimated from the experimental value [PBE (2.12 eV), Expt. (3.2 eV)]. On varying the “Hubbard parameter U” one can reproduce the experimental band gap with PBE + U approach. With U = 6.3 eV, we could reproduce the band gap to be 3.2 eV. However, the energetics of PBE + U to estimate defect formation energy of a single oxygen vacancy at various charge states doesn’t match with respect to advanced hybrid xc-functional HSE06 (see Supplementary Information; Figs S1 and S2). Note that, the default *α* = 0.25 value of HSE06 is unable to reproduce the exact experimental band gap. We find that a proportion of 22% Hartree Fock exchange with 78% PBE exchange produces the experimental band-gap (3.2 eV) for HSE06. In view of this, the accuracy of HSE06 with *α* = 0.22 needs also to be validated. We thus further validate HSE06 xc-functional (with *α* = 0.22) with respect to experimentally observed optical transition of various (X)_O_ (X = N, C, S, Se) (i.e. X substituted at oxygen vacancy defects) in TiO_2_. The optical transitions associated with the process ${{\rm{X}}}_{{\rm{O}}}^{0}+{\rm{h}}\nu \to {{\rm{X}}}_{{\rm{O}}}^{+}+{{\rm{e}}}^{-}$ are shown in (Fig. 2) by forming a configuration co-ordinate diagram. To do this, we have taken 3 different geometries of X_O_ (optimized for charges = 0, +1, +2). Then, we have taken intermediate structure between geometry 1 and geometry 2 (simply by averaging the atomic positions of the geometry 1 and 2). The same is then repeated for the geometry 2 and geometry 3. In this way, we have achieved the 5 intermediate relaxed structure. We then linearly interpolate the relaxed structures in the ground and the constrained state by using five intermediate structures and calculate the formation energies using HSE06 xc-functional (with *α* = 0.22). A parabolic fit of the formation energies of these five intermediate geometries is thus obtained. The same is then repeated for the other charge states (+2 is not shown here). Now the possible transitions are illustrated as shown in Fig. 2. Knowing the optical transitions, the corresponding optical spectra can thus be computed. The details of this state-of-the-art theoretical spectroscopy techniques are explained by Rinke *et al*.^47^. The process involved absorption of photon and emission of electron. The Peak positions are determined by assuming that the atoms in the vicinity of the defect do not have time to relax during the transition from charge state *q* to charge state *q*′ = *q* ± 1(Franck-Condon principle). Thus, the difference in formation energies in charge states *q* and *q*′ gives the absorption and emission energy. The difference in the value of absorption and emission peaks arises due to difference in geometry relaxation for both the charge states *q* and *q*′. The amount of energy lost during relaxation to the new equilibrium position is the Franck-Condon shift. We have found that X_O_ leads to sub-bandgap optical transitions centred at 2.8 eV (442 nm) for N, 2.04 eV (607 nm) for C, 2.32 eV (534 nm) for S and 1.92 eV (645 nm) for Se respectively. These results are in good agreement with the experimental observations in which sizeable absorption occurs at visible region (400–700 nm)^48–51^. Note that, we have also tried the same for Phosphorous substitution at O-site in TiO_2_ anatase. Unfortunately, we have got the response in infra-red region (1.48 eV) (see Supplementary Information; Fig. S6). In view of this, we have not considered it for further study. Therefore, this validates that our HSE06 xc-functional with (*α* = 0.22) is sufficiently accurate to ensure correct energetics at various charge defects in TiO_2_.

### Formation energy of charged defects

We have now considered three types of defects viz. (X)_O_, (XO)_O_ and (X_2_)_O_ with X = N, C, S, Se to understand the most stable defect with specific charge state *q* at a given *T* and ${p}_{{{\rm{O}}}_{2}}$. The explicit dependence on *T* and ${p}_{{{\rm{O}}}_{2}}$ is introduced by employing the concept of *ab initio* atomistic thermodynamics^39^. We address the stability of the substitutional and interstitial configurations of N, C, S, and Se dopants in TiO_2_ by computing formation energies as a function of chemical potentials of the respective species viz. *μ*_O_, *μ*_N_, *μ*_C_, *μ*_S_, *μ*_Se_ as discussed in the following sections. Here the doping is considered as varying the chemical potential of electrons *μ*_e_. In the following sections, we will use ${{\rm{E}}}_{{\rm{f}}}[({\rm{X}}{)}_{{\rm{O}}}^{q}]$, ${{\rm{E}}}_{{\rm{f}}}[({\rm{XO}}{)}_{{\rm{O}}}^{q}]$, ${{\rm{E}}}_{{\rm{f}}}[{({{\rm{X}}}_{2})}_{{\rm{O}}}^{q}]$ as the formation energies of (X)_O_, (XO)_O_ and (X_2_)_O_ related defects at a charge state *q* for X = N, C, S, Se. Similarly, E[XTi_16_O_31_]^*q*^, E[XTi_16_O_32_]^*q*^ and E[X_2_Ti_16_O_31_]^*q*^ are the total DFT energies (HSE06) at a charge state *q* of the respective supercells. E[Ti_16_O_32_]^0^ represents the total DFT energy (HSE06) of the pristine supercell at neutral charge state. E[N_2_] and E[O_2_] are the total DFT (HSE06) energies of N_2_ and O_2_ molecules respectively.

### N-related dopant

The formation energy of (N)_O_ in charge state *q* is given by:

1 $$\begin{array}{rcl}{{\rm{E}}}_{{\rm{f}}}[{({\rm{N}})}_{{\rm{O}}}^{q}] & = & {\rm{E}}{[{{\rm{NTi}}}_{16}{{\rm{O}}}_{31}]}^{q}-{\rm{E}}{[{{\rm{Ti}}}_{16}{{\rm{O}}}_{32}]}^{0}\\  &  & +\,{\mu }_{{\rm{O}}}-{\mu }_{{\rm{N}}}+q({\mu }_{{\rm{e}}}+{\rm{VBM}}+{\rm{\Delta }}{\rm{V}})\end{array}$$

where, *μ*_e_ is the chemical potential of the electron referenced to the valence-band maximum (VBM) of pristine neutral supercell. ΔV accounts for the core level alignment between (Ti_16_O_31_)^*q*^ and (Ti_16_O_32_)^0^.

$${\mu }_{{\rm{N}}}={\rm{\Delta }}{\mu }_{{\rm{N}}}+\frac{1}{2}{\rm{E}}[{{\rm{N}}}_{2}]\,{\rm{and}}\,{\mu }_{{\rm{O}}}={\rm{\Delta }}{\mu }_{{\rm{O}}}+\frac{1}{2}{\rm{E}}[{{\rm{O}}}_{2}]$$

Therefore, Δ*μ*_O_ can be written as a function of $(T,{p}_{{{\rm{O}}}_{2}})$ [see details of this methodology in ref.^52^].

2 $$\begin{array}{ccc}{\rm{\Delta }}{\mu }_{O}(T,{p}_{{O}_{2}}) & = & \frac{1}{2}[-{k}_{B}T\,{\rm{l}}{\rm{n}}[{(\frac{2\pi m}{{h}^{2}})}^{\frac{3}{2}}{({k}_{B}T)}^{\frac{5}{2}}]\\  &  & +\,{k}_{B}T\,{\rm{l}}{\rm{n}}\,{p}_{{O}_{2}}-{k}_{B}T\,{\rm{l}}{\rm{n}}(\frac{8{\pi }^{2}{I}_{A}{k}_{B}T}{{h}^{2}})\\  &  & +\,{k}_{B}T\,{\rm{l}}{\rm{n}}[1-\exp (-\frac{h{\nu }_{OO}}{{k}_{B}T})]\\  &  & -\,{k}_{B}T\,{\rm{l}}{\rm{n}}\,{\mathscr{M}}+{k}_{B}T\,{\rm{l}}{\rm{n}}\,\sigma ]\end{array}$$

where m is the mass, ${\mathcal M}$ is the spin multiplicity, and *σ* is the symmetry number.

3 $$\begin{array}{rcl}{{\rm{E}}}_{{\rm{f}}}[{({\rm{N}})}_{{\rm{O}}}^{q}] & = & {\rm{E}}{[{{\rm{NTi}}}_{16}{{\rm{O}}}_{31}]}^{q}-{\rm{E}}{[{{\rm{Ti}}}_{16}{{\rm{O}}}_{32}]}^{0}+\frac{1}{2}{\rm{E}}[{{\rm{O}}}_{2}]\\  &  & +\,{\rm{\Delta }}{\mu }_{{\rm{O}}}-\frac{1}{2}{\rm{E}}[{{\rm{N}}}_{2}]-{\rm{\Delta }}{\mu }_{{\rm{N}}}+q({\mu }_{{\rm{e}}}+{\rm{VBM}}+{\rm{\Delta }}{\rm{V}})\end{array}$$

The formation energy for interstitial N i.e., (NO)_O_ is given by:

4 $$\begin{array}{rcl}{{\rm{E}}}_{{\rm{f}}}[{({\rm{NO}})}_{{\rm{O}}}^{q}] & = & {\rm{E}}{[{{\rm{NTi}}}_{16}{{\rm{O}}}_{32}]}^{q}-{\rm{E}}{[{{\rm{Ti}}}_{16}{{\rm{O}}}_{32}]}^{0}-\frac{1}{2}{\rm{E}}[{{\rm{N}}}_{2}]\\  &  & -\,{\rm{\Delta }}{\mu }_{{\rm{N}}}+q({\mu }_{{\rm{e}}}+{\rm{VBM}}+{\rm{\Delta }}{\rm{V}})\end{array}$$

As explained above, here ΔV represents the core level alignment between (NTi_16_O_32_)^*q*^ and (Ti_16_O_32_)^0^. Similarly, formation energy for N complex in which interstitial N sharing a lattice site with (N)_O_ i.e., (N_2_)_O_ is given by:

5 $$\begin{array}{rcl}{{\rm{E}}}_{{\rm{f}}}[{({{\rm{N}}}_{2})}_{{\rm{O}}}^{q}] & = & {\rm{E}}{[{{\rm{N}}}_{2}{{\rm{Ti}}}_{16}{{\rm{O}}}_{31}]}^{q}-{\rm{E}}{[{{\rm{Ti}}}_{16}{{\rm{O}}}_{32}]}^{0}+\frac{1}{2}{\rm{E}}[{{\rm{O}}}_{2}]\\  &  & +\,{\rm{\Delta }}{\mu }_{{\rm{O}}}-2{\rm{\Delta }}{\mu }_{{\rm{N}}}-{\rm{E}}[{{\rm{N}}}_{2}]+q({\mu }_{{\rm{e}}}+{\rm{VBM}}+{\rm{\Delta }}{\rm{V}})\end{array}$$

where ΔV is core level alignment between (N_2_Ti_16_O_31_)^*q*^ and (Ti_16_O_32_)^0^. The calculated formation energies of charged and neutral defects depend on the selected values for *μ*_Ti_ and *μ*_O_. The chemical potential of O and Ti must satisfy the stable growth condition for TiO_2_.

6 $${\rm{\Delta }}{\mu }_{{\rm{Ti}}}+2{\rm{\Delta }}{\mu }_{{\rm{O}}}={{\rm{\Delta }}{\rm{H}}}_{{\rm{f}}}({{\rm{TiO}}}_{2})$$

where ΔH_f_(TiO_2_) is the enthalpy of formation for TiO_2_ and its value is −9.73 eV^53^. Next, we present results of the formation energies for O-poor, O-intermediate and O-rich conditions. The O-rich condition corresponds to Δ*μ*_O_ = 0, Δ*μ*_Ti_ = −9.73 eV (obtained from Eq. 6). Under extreme O-poor condition (Ti-rich condition) the growth of Ti_2_O_3_ becomes favorable. As a result, Δ*μ*_Ti_ is estimated by the formation of Ti_2_O_3_.

7 $$2{\rm{\Delta }}{\mu }_{{\rm{Ti}}}+3{\rm{\Delta }}{\mu }_{{\rm{O}}}={{\rm{\Delta }}{\rm{H}}}_{{\rm{f}}}({{\rm{Ti}}}_{2}{{\rm{O}}}_{3})$$

where ΔH_f_(Ti_2_O_3_) is the enthalpy of formation for Ti_2_O_3_ and its value is −15.35 eV^53^. From above two equations (Eqs 6 and 7) we can write Δ*μ*_Ti_ = 2ΔH_f_(Ti_2_O_3_) − 3ΔH_f_(TiO_2_) and Δ*μ*_O_ = −ΔH_f_(Ti_2_O_3_) + 2ΔH_f_(TiO_2_). Solving this we get, under O-poor condition Δ*μ*_Ti_ = −1.51 eV and Δ*μ*_O_ = −4.11 eV. Defect formation energy depends on the chemical potential of Ti, O and the doped impurities. Note that to avoid the precipitation of the host elements, the chemical potential *μ*_X_ must have a constraint that Δ*μ*_X_ ≤ 0. We find that under O-poor conditions (i.e. Ti-rich) Δ*μ*_X_ = 0 and Δ*μ*_O_ = −4.1 eV. Under O-rich conditions Δ*μ*_O_ = 0 and *μ*_N_ is estimated by the formation of N_2_ molecule, i.e., Δ*μ*_N_ = 0; while the limit of the other impurities X (excluding N) (X = C, S, Se) are determined by the formation of the corresponding oxides i.e.,

8 $$x\times {\rm{\Delta }}{\mu }_{{\rm{X}}}+y\times {\rm{\Delta }}{\mu }_{{\rm{O}}}={{\rm{\Delta }}{\rm{H}}}_{{\rm{f}}}({{\rm{X}}}_{{\rm{x}}}{{\rm{O}}}_{{\rm{y}}})$$

The chemical potentials Δ*μ*_X_ for X = C, S, Se are, therefore, determined by formation enthalpy of CO_2_ (ΔH_f_ = −4.07 eV), SO_3_ (ΔH_f_ = −4.101 eV) and SeO_2_ (ΔH_f_ = −2.336 eV) respectively^54^. Note that we have chosen only those oxides which are used as precursor in experiments. With this background, we report now the most stable charged impurities for various non-metals X.

**Figure 3:**
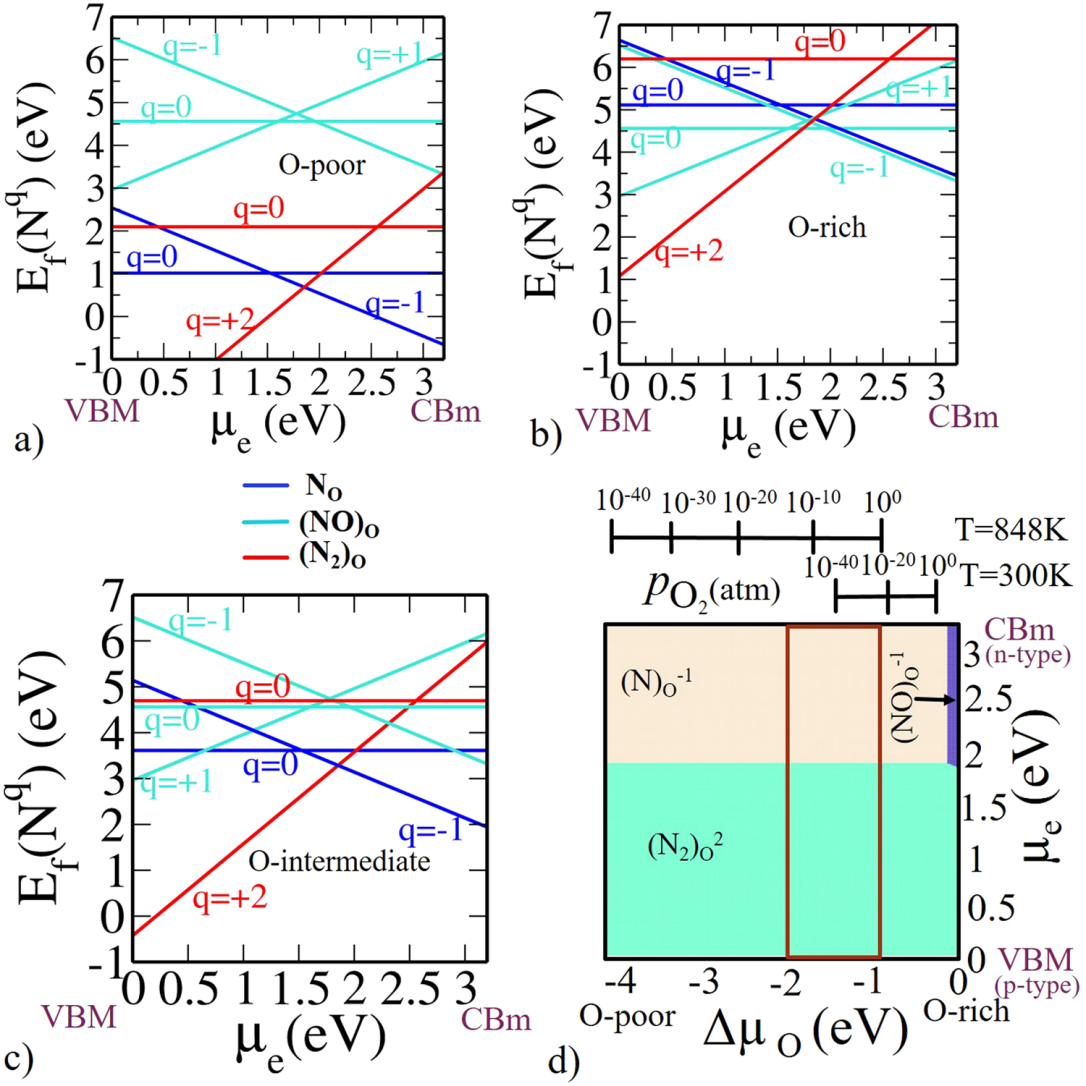
Formation energy for N-related defects in anatase Ti_16_O_32_ (**a**) O-poor limit Δ*μ*_O_ = −4.1 eV and Δ*μ*_N_ = 0 eV. (**b**) O-rich limit Δ*μ*_O_ = 0 eV and Δ*μ*_N_ = 0 eV. (**c**) O-intermediate (Experimental growth condition) Δ*μ*_O_ = −1.5 eV. (**d**) Phase diagram for N-related defects (N)_O_, (NO)_O_, (N_2_)_O_. Here, on x-axis Δ*μ*_O_ is varied in accordance with the corresponding *T* and ${p}_{{{\rm{O}}}_{2}}$. On y-axis *μ*_e_ is varied from valence band maximum to conduction band minimum of the TiO_2_. On z-axis the negative ${{\rm{E}}}_{{\rm{f}}}(T,{p}_{{{\rm{O}}}_{2}})$ values are plotted so that only the most stable phases are visible from the top. The region within the rectangular red lines represents the experimentally relevant conditions at *T* = 848 K with a realistic ${p}_{{{\rm{O}}}_{2}}$ range.

Formation energy is shown as a function of *μ*_*e*_ for (N)_O_, (NO)_O_, (N_2_)_O_ at various charge states for O-poor, O-rich and O-intermediate limit as shown in Fig. 3. As we have explained, O-poor (Fig. 3a) and O-rich (Fig. 3b) represent two of the extreme possible limit of oxygen concentration (Since Δ*μ*_O_ is a function of *T* and ${p}_{{{\rm{O}}}_{2}}$, at a given *T* = 300 K, O-poor and O-rich conditions respectively correspond to a pressure ${p}_{{{\rm{O}}}_{2}}={10}^{-129}$ and 10^9^ atm), while the O-intermediate (see Fig. 3c) represents an experimental condition.

The intersection of the formation energies [e.g. 0/−1, +1/−1, +2/−1 etc.] for the different charge states correspond to the thermodynamic transition levels. Note that N has one electron less than O in the valence shell. Therefore, (N)_O_ in TiO_2_ is expected to act as an acceptor level. It can be stable either in ${({\rm{N}})}_{{\rm{O}}}^{0}$ or ${({\rm{N}})}_{{\rm{O}}}^{-}$ charge states. At a lower *μ*_*e*_ i.e., near valence band maximum (VBM) ${({\rm{N}})}_{{\rm{O}}}^{0}$ is stable and for a larger value of *μ*_*e*_ (i.e., in the upper part near the conduction band minimum (CBm)) ${({\rm{N}})}_{{\rm{O}}}^{-}$ is stable (see Fig. 3). Since, Nitrogen will act as an electron acceptor because it has one electron less than oxygen, it is more feasible at higher values of *μ*_*e*_ i.e., n-type doping. Our key conclusion from calculated formation energies indicate that (N)_O_ is the prevalent defect for *μ*_*e*_ values near CBm for the O-poor and O-intermediate condition. The thermodynamic transition level (0/−1) is located at *μ*_*e*_ = 1.50 eV above the VBM, which indicates that (N)_O_ is a deep acceptor. The formation energies of (N)_O_ under O-poor condition is rather high and it is even higher under O-intermediate and O-rich condition. Therefore (N)_O_ is not suitable for p-type doping in TiO_2_. This result is similar to the behavior of (N)_O_ in ZnO^55^ and SrTiO_3_^56^, indicating that (N)_O_ cannot give p-type conductivity in TiO_2_. To mimic the experimental growth condition, i.e. ${p}_{{{\rm{O}}}_{2}}=2\times {10}^{5}\,{\rm{Torr}}$ and *T* = 575 °C, we have obtained the corresponding value of Δ*μ*_O_ = −1.5 eV using Eq. 2. The stability of (N)_O_ at this value of Δ*μ*_O_ (see Fig. 3c) is well in agreement with previous findings^28^ of Verley *et al*.

In (NO)_O_ defect, N forms a strong bond with a O atom in the TiO_2_ lattice so that in the relaxed configuration interstitial N shares the same site as that of O atom. (NO)_O_ introduces a state in the band gap that is partially occupied with one electron. According to the Pauli’s exclusion principle, maximum two electrons can be filled in one state. Hence, there are possibilities of removal and addition of electrons, resulting in ${({\rm{NO}})}_{{\rm{O}}}^{+}$ and ${({\rm{NO}})}_{{\rm{O}}}^{-}$ charge states. ${({\rm{NO}})}_{{\rm{O}}}^{+}$ is stable for a smaller value of *μ*_*e*_ near VBM (p-type doping) and ${({\rm{NO}})}_{{\rm{O}}}^{-}$ is more significant for a larger value of *μ*_*e*_ near CBm (n-type doping).

We also find that interstitial N can form a complex with (N)_O_ in the form of (N_2_)_O_ split interstitial. (N_2_)_O_ has a deficiency of one electron than (NO)_O_, so (N_2_)_O_ should be stable as ${({{\rm{N}}}_{2})}_{{\rm{O}}}^{+2}$ or ${({{\rm{N}}}_{2})}_{{\rm{O}}}^{0}$. ${({{\rm{N}}}_{2})}_{{\rm{O}}}^{+2}$ is energetically preferable for the lower part of the band gap near VBM and ${({{\rm{N}}}_{2})}_{{\rm{O}}}^{0}$ is preferable for the upper part of the band gap near CBm.

Now, we can summarize the whole information from a phase diagram where negative z-axis corresponds to formation energy as shown in Fig. 3d. Since we are visualizing from the top, we are limited to see only the most negative values i.e., we see only the most stable phases. On x-axis, we have Δ*μ*_O_ varied from O-poor to O-rich conditions. On y-axis, we have *μ*_*e*_ referenced to VBM. Among all the three possible configurations, we observed from Fig. 3d, ${({{\rm{N}}}_{2})}_{{\rm{O}}}^{+2}$ is the most stable defect state near VBM (p-type doping). In n-type doping, ${({\rm{N}})}_{{\rm{O}}}^{-1}$ is the most stable defect state near CBm under both O-poor and O-intermediate while under O-rich ${({\rm{NO}})}_{{\rm{O}}}^{-1}$ is stable. The rectangular box is showing the experimentally feasible region at T = 848 K with a viable pressure range.

### C-related dopant

Similarly for Carbon (C) related defects, we have considered three configurations: Carbon substituted oxygen (C)_O_, Carbon as interstitial (CO)_O_, Interstitial carbon sharing a lattice site with (C)_O_ forming (C_2_)_O_. The formation energy can be calculated in a similar way for all the three configurations using either C or CO_2_ as a precursor. In either case we have checked that the results are same. To use CO_2_ as the precursor, we have used the following formula to estimate E[C]:

9 $${\rm{E}}[{\rm{C}}]={\rm{E}}[{{\rm{CO}}}_{2}]-{\rm{E}}[{{\rm{O}}}_{2}]-{{\rm{\Delta }}{\rm{H}}}_{{\rm{f}}}[{{\rm{CO}}}_{2}]$$

**Figure 4:**
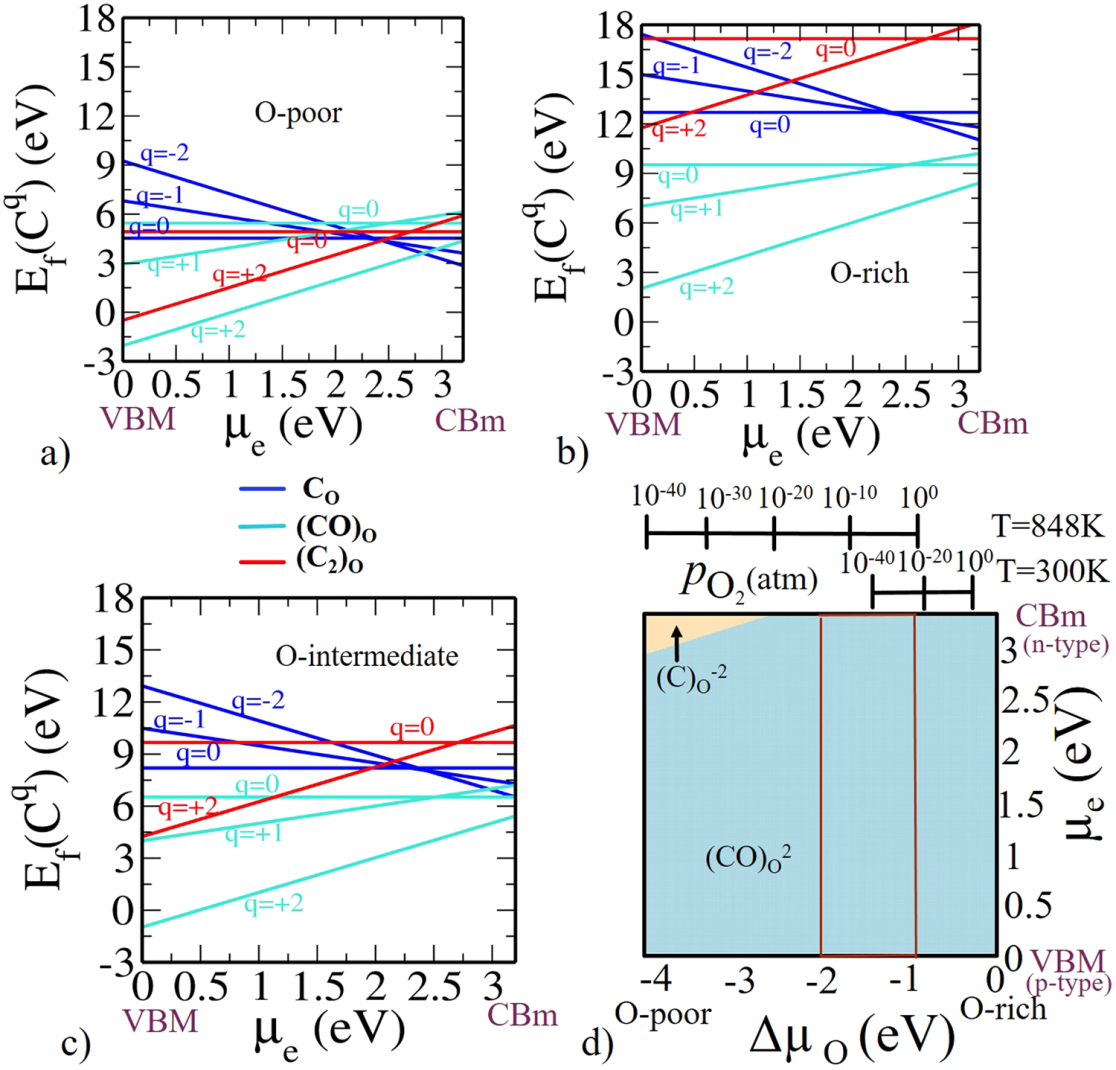
Formation energy for C-related defects in anatase Ti_16_O_32_ (**a**) O-poor limit Δ*μ*_O_ = −4.1 eV and Δ*μ*_C_ = 0 eV. (**b**) O-rich limit Δ*μ*_O_ = 0 eV and Δ*μ*_C_ = −4.07 eV. (**c**) O-intermediate (Experimental growth condition) Δ*μ*_O_ = −1.5 eV. (**d**) Phase diagram for C-related defects (C)_O_, (CO)_O_, (C_2_)_O_. The region within the rectangular red lines represents the experimentally relevant conditions at *T* = 848 K with a realistic ${p}_{{{\rm{O}}}_{2}}$ range.

The same can be calculated directly from DFT total energy from a supercell of C_8_. The equations related to the formation energy of C related defects in charge state *q* are given in Supplementary Information. In Fig. 4 we have shown the variation of formation energy as a function of *μ*_*e*_ for (C)_O_, (CO)_O_, (C_2_)_O_ at various charge states under O-poor, O-rich and O-intermediate conditions. We know from the electronic configuration of C that it has two electrons less than O in the valence shell. Therefore, (C)_O_ in TiO_2_ is expected to act as an acceptor and be stable in either the ${({\rm{C}})}_{{\rm{O}}}^{0}$, ${({\rm{C}})}_{{\rm{O}}}^{-1}$ and ${({\rm{C}})}_{{\rm{O}}}^{-2}$ charge states. In (C)_O_ defect, at a lower values of *μ*_*e*_ i.e., near VBM ${({\rm{C}})}_{{\rm{O}}}^{0}$ is stable and for larger values of *μ*_*e*_ (i.e., in the upper part near the CBm) ${({\rm{C}})}_{{\rm{O}}}^{-2}$ is stable (see Fig. 4) for all the three conditions. Our main concern is near CBm as it is an acceptor dopant.

Note that for interstitial C in TiO_2_ i.e., (CO)_O_ induces several localized occupied states in the band gap. Hence it is stable either in neutral charge state ${({\rm{CO}})}_{{\rm{O}}}^{0}$ or in +2 charge state ${({\rm{CO}})}_{{\rm{O}}}^{+2}$. From Fig. 4, it is clear that ${({\rm{CO}})}_{{\rm{O}}}^{+2}$ is stable for all values of *μ*_*e*_ for all the three conditions. Interstitial C can form a complex with (C)_O_ in the form of (C_2_)_O_. (C_2_)_O_ has two electrons fewer than (CO)_O_, and we thus find it to be stable as ${({{\rm{C}}}_{2})}_{{\rm{O}}}^{+2}$. In this way we have chosen the favorable charges for our calculations that can be expected on the basis of fundamental chemical intuition. DFT energetics further validate the same. ${({{\rm{C}}}_{2})}_{{\rm{O}}}^{+2}$ is energetically preferable for lower part of the band gap and ${({{\rm{C}}}_{2})}_{{\rm{O}}}^{0}$ is preferable for upper part of the band gap. Our key conclusion from calculated formation energies indicate that at O-poor condition, (C)_O_ is the prevalent defect for *μ*_*e*_ values near CBm while for O-rich and O-intermediate conditions, C has predominant preference for occupying the interstitial site (CO)_O_. The result regarding stability of configuration (either substitutional or interstitial) is in agreement with previous finding of Ghosh *et al*.^11^ but with charge state −2 and +2 respectively (see the phase diagram as in Fig. 4d). From this phase diagram, under O-poor condition ${({\rm{CO}})}_{{\rm{O}}}^{+2}$ is energetically preferable in lower part of the band gap (p-type doping) and ${({\rm{C}})}_{{\rm{O}}}^{-2}$ is predominant near CBm (n-type doping), while under O-rich condition ${({\rm{CO}})}_{{\rm{O}}}^{+2}$ is preferable for all values of *μ*_e_. The rectangular box is showing the experimentally feasible region at T = 848 K with a realistic oxygen partial pressure ${p}_{{{\rm{O}}}_{2}}$ range.

### S-related dopant

Next, we have addressed Sulfur (S) related defects, where we have considered the same three configurations: Sulfur substituted oxygen (S)_O_, Sulfur as interstitial (SO)_O_, and interstitial Sulfur sharing a lattice site with (S)_O_ forming (S_2_)_O_. The formation energy can be calculated in a similar way for all the three configurations using either S or SO_3_ as a precursor. In either case we have verified that the results are same. To use SO_3_ as a precursor, we have used the following formula to estimate E(S):

10 $${\rm{E}}[{\rm{S}}]={\rm{E}}[{{\rm{SO}}}_{3}]-\frac{3}{2}{\rm{E}}[{{\rm{O}}}_{2}]-{{\rm{\Delta }}{\rm{H}}}_{{\rm{f}}}[{{\rm{SO}}}_{3}]$$

**Figure 5:**
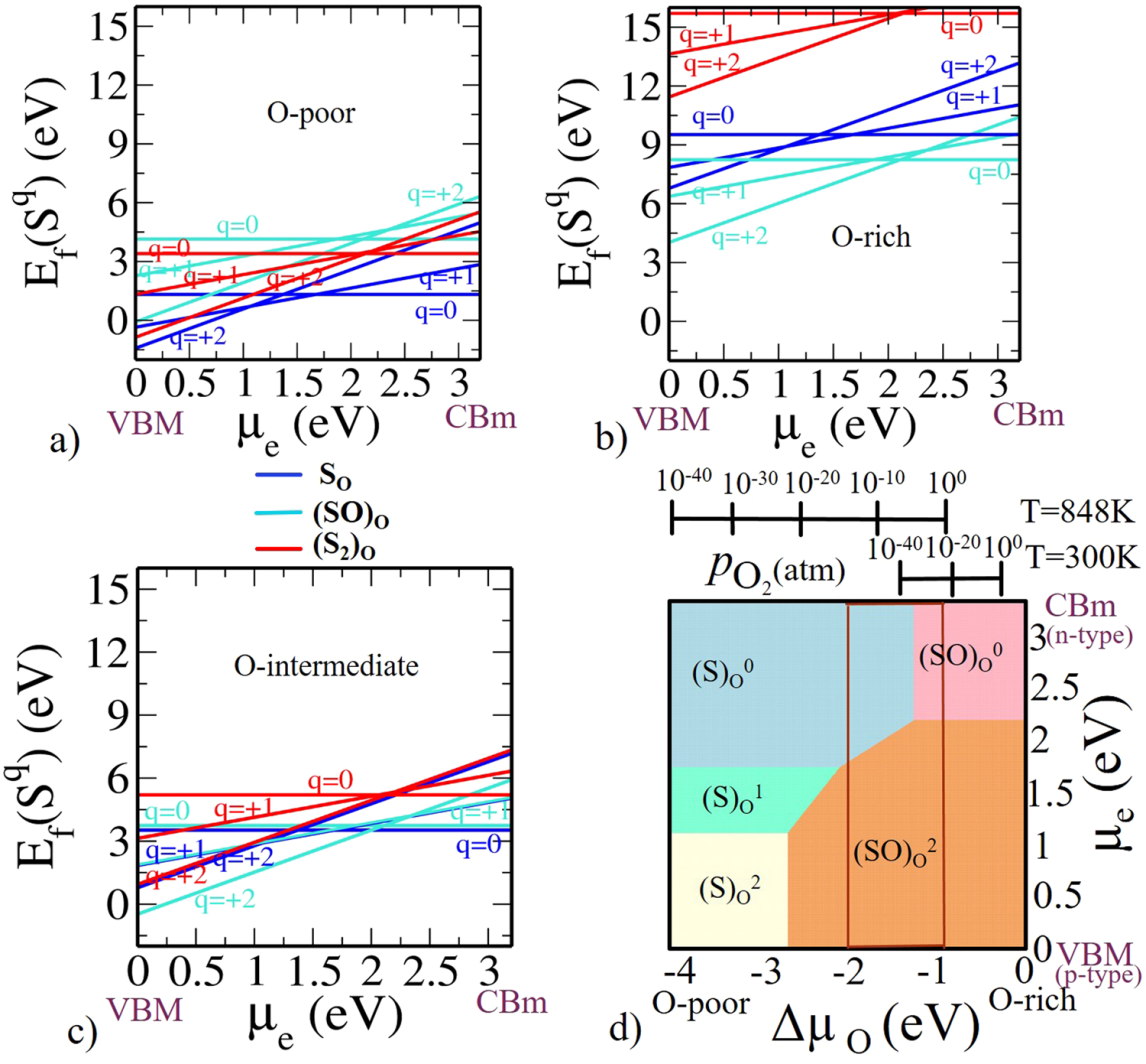
Formation energy for S-related defects in anatase Ti_16_O_32_ (**a**) O-poor limit Δ*μ*_O_ = −4.1 eV and Δ*μ*_S_ = 0 eV. (**b**) O-rich limit Δ*μ*_O_ = 0 eV and Δ*μ*_S_ = −4.101 eV. (**c**) O-intermediate (Experimental growth condition) Δ*μ*_O_ = −1.5 eV. (**d**) Phase diagram for S-related defects (S)_O_, (SO)_O_, (S_2_)_O_. The region within the rectangular red lines represents the experimentally relevant conditions at *T* = 848 K with a realistic ${p}_{{{\rm{O}}}_{2}}$ range.

The same can also be calculated directly from DFT total energy from a supercell of S_8_ (see equations in Supplementary Information). In Fig. 5, the formation energy is shown as a function of *μ*_*e*_ for (S)_O_, (SO)_O_, (S_2_)_O_ at various charge states (viz. 0/+1/+2/−1/−2) for O-poor, O-rich and O-intermediate limits. (S)_O_ has the same number of electrons in the outermost shell as oxygen. Insertion of S into the TiO_2_ lattice is difficult to achieve due to its larger ionic radius. However, Periyat *et al*. have reported S-doped TiO_2_ through modification of titanium isopropoxide with sulphuric acid^57^. Note that the insertion of cationic sulfur (positive charge) is chemically favorable over the anionic sulfur (negative charge)^58^. Our findings are quite in agreement with this observation. We have found that positive charges (viz. 0/+1/+2) are more favorable than negative charges. Hence, it will act as an electron donor dopant, which is more feasible at lower values of *μ*_*e*_ i.e., p-type doping. In (S)_O_ defect, at a lower values of *μ*_*e*_ i.e., near VBM ${({\rm{S}})}_{{\rm{O}}}^{+2}$ is stable and for larger values of *μ*_*e*_ (i.e., in the upper part near CBm) ${({\rm{S}})}_{{\rm{O}}}^{0}$ is stable (see Fig. 5) for all the three conditions. In (SO)_O_ defect, it is clear from Fig. 5 that ${({\rm{SO}})}_{{\rm{O}}}^{+2}$ is stable for lower values of *μ*_*e*_ and ${({\rm{SO}})}_{{\rm{O}}}^{0}$ is stable for higher values of *μ*_*e*_. Interstitial S complex (S_2_)_O_ has same number of electrons as (SO)_O_. Consequently, (S_2_)_O_ preferred the same charge state as that by (SO)_O_. From Fig. 5 ${({{\rm{S}}}_{2})}_{{\rm{O}}}^{+2}$ is energetically preferable for lower part of the band gap and ${({{\rm{S}}}_{2})}_{{\rm{O}}}^{0}$ is preferable for upper part of the band gap. We can summarize them all into a phase diagram as shown in Fig. 5d. Under O-poor condition, ${({\rm{S}})}_{{\rm{O}}}^{+2}$ is energetically favorable for lower values of *μ*_*e*_ and ${({\rm{S}})}_{{\rm{O}}}^{0}$ is preferable for upper values of *μ*_*e*_ i.e., near CBm. Under O-rich condition, ${({\rm{SO}})}_{{\rm{O}}}^{+2}$ is energetically preferable for lower part of the band gap and ${({\rm{SO}})}_{{\rm{O}}}^{0}$ becomes prominent for upper part of the band gap. These findings indicate that the synthesis of substitutional S (S)_O_ may be easier than that of interstitial S (SO)_O_ under the O-poor condition because of low formation energy. On the contrary, under the O-rich condition (less Ti atoms), the calculated formation energy shows that interstitial S (SO)_O_ is more prevalent defect than other configurations.

### Se-related dopant

**Figure 6:**
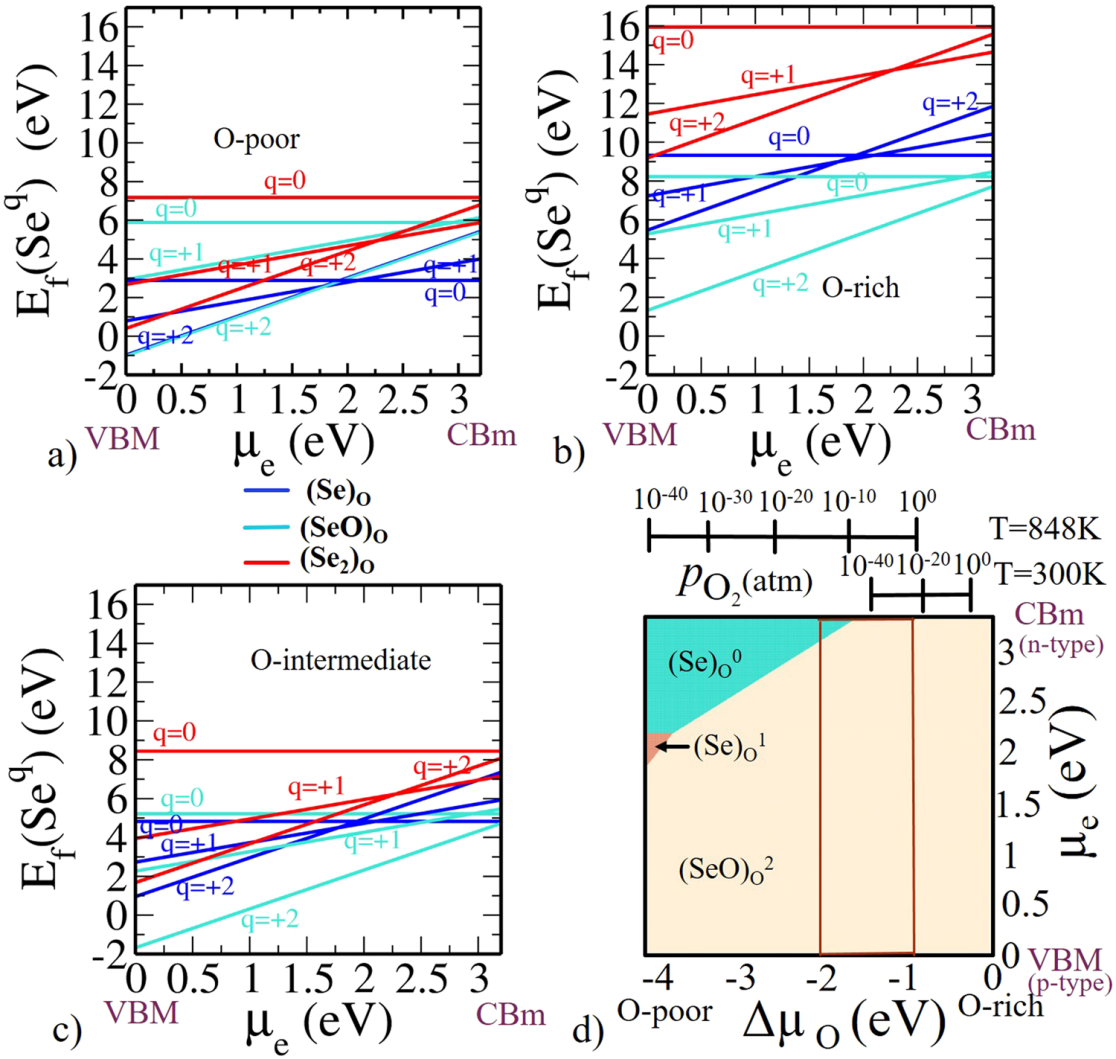
Formation energy for Se-related defects in anatase Ti_16_O_32_ (**a**) O-poor limit Δ*μ*_O_ = −4.1 eV and Δ*μ*_Se_ = 0 eV. (**b**) O-rich limit Δ*μ*_O_ = 0 eV and Δ*μ*_Se_ = −2.336 eV. (**c**) O-intermediate (Experimental growth condition) Δ*μ*_O_ = −1.5 eV. (**d**) Phase diagram for Se-related defects (Se)_O_, (SeO)_O_, (Se_2_)_O_. The region within the rectangular red lines represents the experimentally relevant conditions at *T* = 848 K with a realistic ${p}_{{{\rm{O}}}_{2}}$ range.

The formation energy formula for Selenium related defects for all the three configurations using SeO_2_ as a precursor is given in Supplementary Information. The calculated Formation energy is shown in Fig. 6 as a function of *μ*_*e*_ for (Se)_O_, (SeO)_O_, (Se_2_)_O_ at various charge states for O-poor, O-rich and O-intermediate limits.

Like S, Se has same number of electrons in the outermost shell as that of oxygen. Selenium possessing both cationic and anionic doping behavior. Thus, it can potentially provide electron trapping centers, which results in inhibition of their recombination with photo-generated holes. Hence, it can act as an electron donor as well as an electron acceptor dopant. From the calculated formation energies we have found that positive charges (viz. 0/+1/+2) are more favorable than negative charges. Hence, it will act as electron donor dopant, which is more feasible at lower values of *μ*_*e*_ i.e., p-type doping. In (Se)_O_ defect, at lower values of *μ*_*e*_ i.e., near VBM ${({\rm{Se}})}_{{\rm{O}}}^{+2}$ is stable and for larger values of *μ*_*e*_ (i.e., in the upper part near the CBm) ${({\rm{Se}})}_{{\rm{O}}}^{0}$ is stable (see Fig. 6) under any equilibrium growth condition. In (SeO)_O_ defect, it is clear from Fig. 6 that ${({\rm{SeO}})}_{{\rm{O}}}^{+2}$ is stable for all values of *μ*_*e*_ for all the three conditions. Interstitial S complex (Se_2_)_O_ has same number of electrons as (SeO)_O_. Consequently, (Se_2_)_O_ preferred the same charge state as preferred by (SeO)_O_. ${({{\rm{Se}}}_{2})}_{{\rm{O}}}^{+2}$ is energetically preferable for lower part of the band gap and ${({{\rm{Se}}}_{2})}_{{\rm{O}}}^{+1}$ is preferable for upper part of the band gap. Phase diagram shown in Fig. 6(d) summarize the whole information briefly. It states that under O-poor condition, ${({\rm{SeO}})}_{{\rm{O}}}^{+2}$ is energetically favorable for lower part of band gap and ${({\rm{Se}})}_{{\rm{O}}}^{0}$ is preferable for upper part i.e near CBm. Under O-rich condition, ${({\rm{SeO}})}_{{\rm{O}}}^{+2}$ is energetically preferable for all values of *μ*_*e*_ throughout the band gap.

Note that, from all the above explanation it is cleared that, in order to stabilize the system in case of doping, we need to supply external charges into the system. This means if we assume of having reservoir of electrons (from where the dopant can trap one electron), the dopant will be stabilized at the defect site. This is as good as claiming the presence of having several oxygen vacancies in the lattice with uncompensated electrons and those electrons can be trapped by the external dopant. Thus we can adapt either of the approaches i.e. (1) addition of external charge to the dopant or (2) explicit presence of oxygen vacancy for the neutral dopants. In the literatures, people have adopted the methodology in which charge compensation are done to the (neutral) dopant by creating O-vacancies^24,25,59–62^. However, here we have adopted a different approach, where the charge compensation are performed by adding or removing the extra electrons/holes to the dopant (charged defects). Both the approaches yield to the same conclusion as they are doing effectively the same charge compensation at the defect site and therefore our results are well in agreement with the previous findings of the Pacchioni’s group^24,25,59–62^. For further details see Supplementary Information; (Figs S3 and S4).

### Underlying reasons for extra stability of a specific charged defects

**Figure 7:**
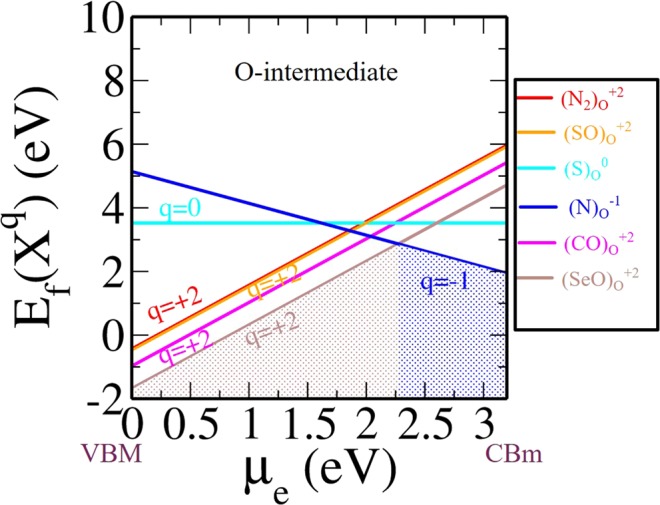
Formation energy for X-related defects that include the most stable defects obtained from 3D phase diagram in anatase Ti_16_O_32_ at O-intermediate condition.

In order to determine the most abundant non-metal dopants in TiO_2_, we have compared the thermodynamic stability of all the most stable individual non-metal defects cases (viz. N, C, S, Se at various charge states as obtained from respective 3-D phase diagrams) at O-intermediate conditions (see Fig. 7). We see the most relevant defects are ${({\rm{SeO}})}_{{\rm{O}}}^{+2}$, ${({\rm{CO}})}_{{\rm{O}}}^{+2}$, ${({\rm{SO}})}_{{\rm{O}}}^{+2}$, ${({{\rm{N}}}_{2})}_{{\rm{O}}}^{+2}$, ${({\rm{S}})}_{{\rm{O}}}^{0}$ and ${({\rm{N}})}_{{\rm{O}}}^{-1}$. It is shown in Fig. 7 that at O-intermediate condition, ${({\rm{SeO}})}_{{\rm{O}}}^{+2}$ is the most stable defect at the lower value of *μ*_*e*_, whereas ${({\rm{N}})}_{{\rm{O}}}^{-1}$ is the only stable defect at the higher value of *μ*_*e*_. A similar study at O-rich and O-poor condition is given in (see Supplementary Information; Fig. S5).

It’s interesting to understand the underlying reason for this enhanced stability of ${({\rm{SeO}})}_{{\rm{O}}}^{+2}$ over ${({{\rm{N}}}_{2})}_{{\rm{O}}}^{+2}$, ${({\rm{CO}})}_{{\rm{O}}}^{+2}$, ${({\rm{SO}})}_{{\rm{O}}}^{+2}$ in pristine TiO_2_ in the p-type doping condition. Therefore, the enhanced stability of ${({\rm{SeO}})}_{{\rm{O}}}^{+2}$ is carefully further analyzed quantitatively. For this purpose, we have decomposed the overall defect formation energy into two parts: (i) bond-making/breaking energy (Δ$\varepsilon$) and (ii) charge carrier trapping energy (Δ$\zeta$). The combined effect of these two competing factors decides which defect configurations will get more stabilized over other configurations. Now Δ$\varepsilon$ is given by:

11 $${\rm{\Delta }}\varepsilon ={{\rm{E}}}_{{\rm{defect}}}^{q}-{{\rm{E}}}_{{\rm{pristine}}}^{{\rm{0}}}\pm {{\rm{E}}}_{{\rm{constituents}}}\pm \sum _{i}\,{n}_{i}\times {\mu }_{i}$$

**Figure 8:**
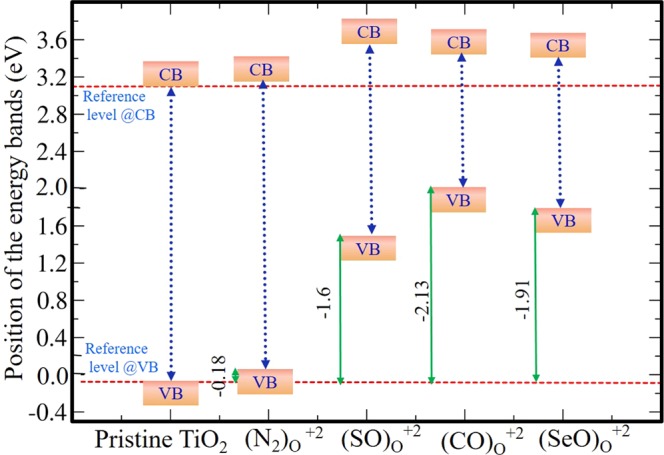
Band positions of most stable charged defect configurations.

Note that *μ*_e_ is varied from VBM (i.e. highest possible p-type doping) to the CBm (i.e. highest possible n-type doping). Here to understand the stability at a given *μ*_*e*_, we have fixed our *μ*_*e*_ at VBM of pristine TiO_2_ at charge state 0. The VBM of pristine Ti_16_O_32_ is taken as a reference for calculating charge carrier trapping energy. Therefore, the charge carrier trapping energy i.e., Δ$\zeta$ is given by (see Fig. 8):

12 $${\rm{\Delta }}\zeta ={\rm{VBM}}{({{\rm{TiO}}}_{2})}^{0}-{\rm{VBM}}{({\rm{defect}})}^{q}$$

It should be mentioned here that Eq. 12 is explicitly for p-type doping. Note that, for n-type doping one needs to set a different *μ*_*e*_ as reference level in the n-type region (viz. CBm(TiO_2_)^0^). From Table 1, we see that Δ$\varepsilon$ is of comparable order for ${({{\rm{N}}}_{2})}_{{\rm{O}}}^{+2}$, ${({\rm{SO}})}_{{\rm{O}}}^{+2}$ and ${({\rm{CO}})}_{{\rm{O}}}^{+2}$, whereas this is quite low for ${({\rm{SeO}})}_{{\rm{O}}}^{+2}$. However a favourable Δ$\zeta$ further stabilizes ${({\rm{CO}})}_{{\rm{O}}}^{+2}$ and ${({\rm{SeO}})}_{{\rm{O}}}^{+2}$ defects in O-intermediate condition. While comparing these two defects viz. ${({\rm{SeO}})}_{{\rm{O}}}^{+2}$ and ${({\rm{CO}})}_{{\rm{O}}}^{+2}$, we see that the former has more negative Δ$\varepsilon$ value than the latter, whereas the trend is slightly reversed for Δ$\zeta$. However, the sum of Δ$\zeta$ and Δ$\varepsilon$ results a more negative value for ${({\rm{SeO}})}_{{\rm{O}}}^{+2}$ as compared to other stable configurations in O-intermediate region with p-type doping condition. Therefore, this concludes that both the bond-making/breaking energy and charge carrier trapping energy play an important role in providing enhanced stability to ${({\rm{SeO}})}_{{\rm{O}}}^{+2}$ defect configuration in TiO_2_. For n-type region only one stable defect is present, which is ${({\rm{N}})}_{{\rm{O}}}^{-1}$. A similar explanation can also be drawn for the stable compositions at other environmental conditions viz. O-poor and O-rich as shown in (see Supplementary Information; Fig. S5).

**Table 1:** Charge carrier trapping energy (Δ*ζ*) and bond-making/breaking energy (Δ*ε*).

Stable configurations in O-intermediate(Z)	Δ*ζ*	Δ*ε*	Δ*ζ* + Δ*ε*
${({{\rm{N}}}_{2})}_{{\rm{O}}}^{+2}$	−0.18	−0.203	−0.383
${({\rm{SO}})}_{{\rm{O}}}^{+2}$	−1.60	−0.256	−1.86
${({\rm{CO}})}_{{\rm{O}}}^{+2}$	−2.13	−0.754	−2.88
${({\rm{SeO}})}_{{\rm{O}}}^{+2}$	−1.91	−1.46	−3.37

## Conclusion

In summary, we have presented an exhaustive study to understand the most stable non-metal related defect in bulk form of anatase TiO_2_ as a function of charge at a realistic condition. As a first step, we have validated our DFT functionals, where we find that involvement of hybrid functional HSE06 is essential and semi-local functional and its improved variants are not sufficient even to address this problem qualitatively. We further notice that the *α* parameter of HSE06 needs to be adjusted to 0.22 so as to correctly predict the experimental bandgap of 3.2 eV and the optical transitions as found in experiments for different (X)_O_ related defects (X = N, C, S, Se). Depending on the oxygen-concentration, we have analyzed the stability of different configurations at three different environmental conditions at a given *T* and ${p}_{{{\rm{O}}}_{2}}$ i.e. O-rich, O-poor and O-intermediate to mimic all possible experimental growth conditions. Three different defect configurations are considered viz. X substituted O (X)_O_, X as interstitial (XO)_O_ and X as interstitial sharing a lattice site with X_O_, (X_2_)_O_. We have found that substitutional N_O_ and (N_2_)_O_ are energetically favorable in TiO_2_ under most of the equilibrium growth conditions when the material is n-type and p-type doped, respectively. On the other hand, for C-related defects, C has a predominant preference for occupying the interstitial site (CO)_O_. For S-related defects, S substitutional (S_O_) is dominant under the O-poor condition, whereas under O-rich condition, S interstitial (SO)_O_ becomes more stable. And finally, for Se-related defects Se interstitial (SeO)_O_ is prevalent defect under most of the doping conditions, in contrast to Se substitution (Se)_O_, which is slightly favored only at the n-type doped condition under O-poor condition. Finally, we have provided a comparative stability analysis of all the individual stable doped configurations in TiO_2_ at O-intermediate conditions. We conclude that for p-type doping ${({\rm{SeO}})}_{{\rm{O}}}^{+2}$ is the most abundant defect, whereas for n-type doping it’s ${({\rm{N}})}_{{\rm{O}}}^{-1}$. The enhanced stability is also carefully analyzed quantitatively by including the individual effect of bond-making/breaking and the charge carrier trapping energies.

## Computational Details

We have performed the DFT calculations with PAW pseudopotential method^63^ as implemented in Vienna *ab initio* simulation package (VASP)^64^. The supercell, consisting 48 atoms, is constructed by 2 × 2 × 1 replication of the tetragonal TiO_2_ unit cell (space group number:141, I41/amd). The supercell size ensures enough spatial separation between the periodic images of the doped impurities under periodic boundary conditions. We have explicitly checked this by making a single O-vacancy to be fully localized inside the cell. To ensure the supercell size convergence, test calculations using 96-atom supercell have also been performed for the case of O-vacancy. The results are consistent with that obtained from a 48-atom supercell. However, a drawback of limited system sizes is that the dopant concentrations are artificially higher (perhaps two to three times) than the experimental case^11^. In order to ensure that our findings are not just an artifact of DFT functionals, we have used a number of exchange and correlation (xc) functionals viz. generalized gradient approximation (GGA) with PBE^65^, GGA + U^66^ (where U is Hubbard parameter) and hybrid functional HSE06^67,68^. The results given here are from HSE06 xc-functional. The performance of the other xc-functionals are discussed in detail in Supplementary Information; Figs S1 and S2. All the structures are fully relaxed (atomic position) upto 0.001 eV/Å force minimization using conjugate gradient minimization with 4 × 4 × 2 K-mesh. For electronic structure energy calculations, the brillouin zone is sampled with a 8 × 8 × 4 Monkhorst-Pack^69^ K-mesh with 0.01 meV energy tolerance. In all our calculations, the plane wave energy cut-off is set to 600 eV.

## Supplementary information

Supplementary Information
