# Supplementary material for: Stability of non-metal dopants to tune the photo-absorption of TiO2 at realistic temperatures and oxygen partial pressures: A hybrid DFT study
Source: Sci Rep. 2019 Aug 6;9:11427. doi: 10.1038/s41598-019-47710-7 (PMC6684643; doi:10.1038/s41598-019-47710-7)
Supplement: Supplementary file 1 — Supplementary Information [file 41598_2019_47710_MOESM1_ESM.pdf]

Supplementary Information
"Stability of non-metal dopants to tune the photo-absorption of TiO_2 at realistic temperatures and oxygen partial pressures: A hybrid DFT study"

Pooja Basera*, Shikha Saini, Ekta Arora, Arunima Singh, Manish Kumar and Saswata Bhattacharya*
Department of Physics, Indian Institute of Technology Delhi, New Delhi 110016 India
*Corresponding author's email: Pooja.Basera@physics.iitd.ac.in [PB]
*saswata@physics.iitd.ac.in [SB]

Validation of DFT functionals: Formation energy of single O-vacancy (□)

To ensure that our findings are not an artifact of the chosen treatment of the DFT exchange and correlation (xc) functional, we have first thoroughly benchmarked the xc-functionals. We have noted that using PBE, the band gap of TiO_2 is quite underestimated from the experimental value [PBE (2.12 eV), Expt. (3.2 eV)]. Thus we have employed GGA+U approach, where the Hubbard parameter U is varied to get a good agreement with the experimental band gap. The experimental band gap value (3.2 eV) can be reproduced when U = 6.3 eV. However, despite we have a nice match of band gap on inclusion of suitable U, the accuracy of this GGA+U energetics needs to be thoroughly benchmarked. In order to understand defect levels and charge localization, to produce the correct ground state total energy (at various charge states) is an indispensable condition [1]. We have, therefore, calculated the formation energy $[\mathrm{E_f}(\Box)^q]$ of a charged (q) defect for a single oxygen vacancy (□) in 48 atom supercell $[\mathrm{Ti_{16}O_{32}}]$ of TiO_2 using both GGA (PBE) and GGA+U [see Figure S1]. The $\mathrm{E_f}(\Box)^q$ is calculated as below:

$$\mathrm{E_f}(\Box)^q = \mathrm{E_{tot}}(\mathrm{Ti_{16}O_{31}})^q - \mathrm{E_{tot}}(\mathrm{Ti_{16}O_{32}})^0 + \frac{1}{2}\mathrm{E_{tot}}(\mathrm{O_2}) + q(\mu_\mathrm{e} + \mathrm{VBM} + \Delta\mathrm{V}) \quad (1)$$

Here, $\mathrm{E_{tot}}(\mathrm{Ti_{16}O_{31}})^q$ and $\mathrm{E_{tot}}(\mathrm{Ti_{16}O_{32}})^0$ are the total energies of the supercell with one O-vacancy at charge state q and neutral pristine respectively. $\mathrm{E}(\mathrm{O_2})$ is the total energy of one oxygen molecule. μ_e is the chemical potential of the electron referenced to the valence-band maximum (VBM) of pristine neutral supercell. ΔV accounts for the core level alignment between $(\mathrm{Ti_{16}O_{31}})^q$ and $(\mathrm{Ti_{16}O_{32}})^0$.
We find from Figure S1 that using PBE $q = +2$ is stable near valence band, while $q = -2$ is stable near conduction band. On inclusion of U parameter, the situation is similar, except at the middle position where the two plots differ. In order to cross check if this correctly reproduces the energetics, we have employed hybrid functionals HSE06 [2, 3]. The HSE06 method divides the exchange potential into short range and long range parts. The short range part [SR] are described as a fraction of non-local Hartree-Fock exchange potential mixed with the GGA exchange potential of PBE. The long range part [LR] and the correlation potential are described by the PBE (DFT) functional.

$$\mathrm{E_{xc}^{HSE06}} = \alpha \mathrm{E_{xc}^{HF}}(\omega)[\mathrm{SR}] + (1-\alpha)\mathrm{E_{xc}^{DFT}}(\omega)[\mathrm{SR}] + \mathrm{E_{c}^{DFT}}(\omega)[\mathrm{LR}] \quad (2)$$

Where α is the mixing coefficient and ω is the screening length. We find that a proportion of 22% HF exchange with 78% PBE exchange produces the experimental band-gap (3.2 eV) [see Figure S2a]. Hence, in our case α is set to 0.22 and ω is 0.11 bohr^{-1}. Note that, this type of screened hybrid functionals [HSE06] are very advanced to determine the energetics correctly compared to unscreened hybrid functional [4].

Next, we have computed the defect formation energies for different charge states using HSE06 fuctional as shown in Figure S2b. Clearly comparing Figure S1 and Figure S2b, we see that defect formation energy differs in those two plots if the material is n-type doped (see the region near conduction band). In this region according to PBE (or GGA+U), -2 charge state is the most stable one (Figure S1), while using HSE06 +2 is the only stable charge state for one charged O-□ irrespective of any type of doping (Figure S1). Therefore, this validates that for this system, a semi-local functionals e.g. PBE (or its improved variants e.g. GGA+U) is not sufficiently accurate even to draw qualitative informations and hybrid functional HSE06 is essential to estimate a correct formation energy [5, 6]. In view of this, we report the rest of our results using HSE06 with $\alpha = 0.22$.

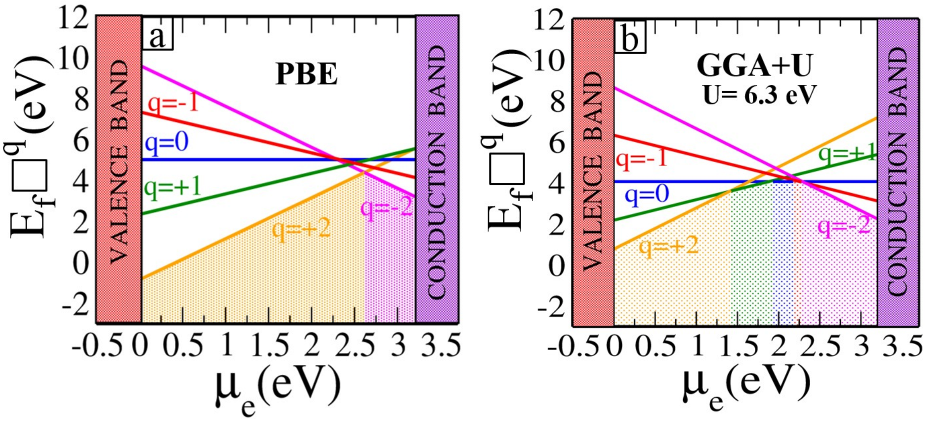

Figure S1: The formation energy of single oxygen vacancy (O-□) as a function of electron chemical potential μ_e for different charge states q (a) Using PBE functional. (b) Using GGA+U functional. Colored areas are for the guideline to identify which charge state is the most stable in the vicinity of valence and conduction band.

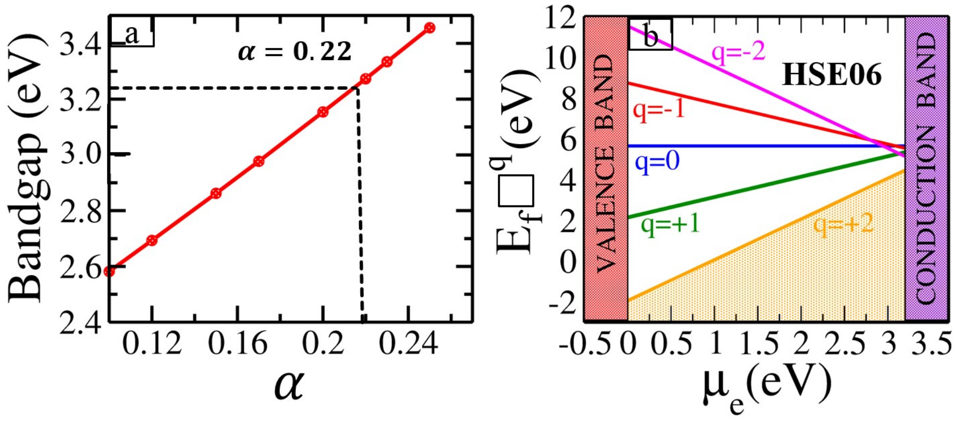

Figure S2: (a) Band gap versus mixing parameter α. (b) Formation energy of a single oxygen vacancy (O-□) as a function of electron chemical potential μ_e for different charge states q using HSE06 functional.

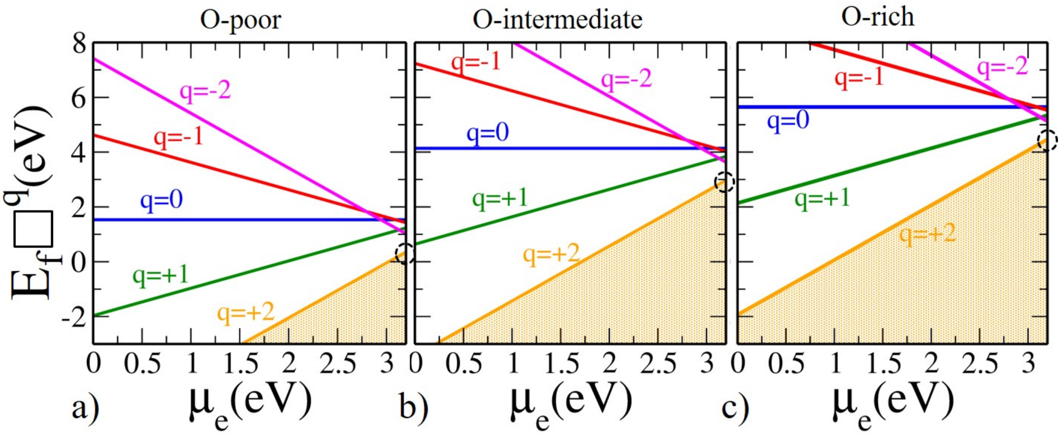

Figure S3: Formation energy plot for single O-vacancy at a) O-poor b) O-intermediate c) O-rich conditions..

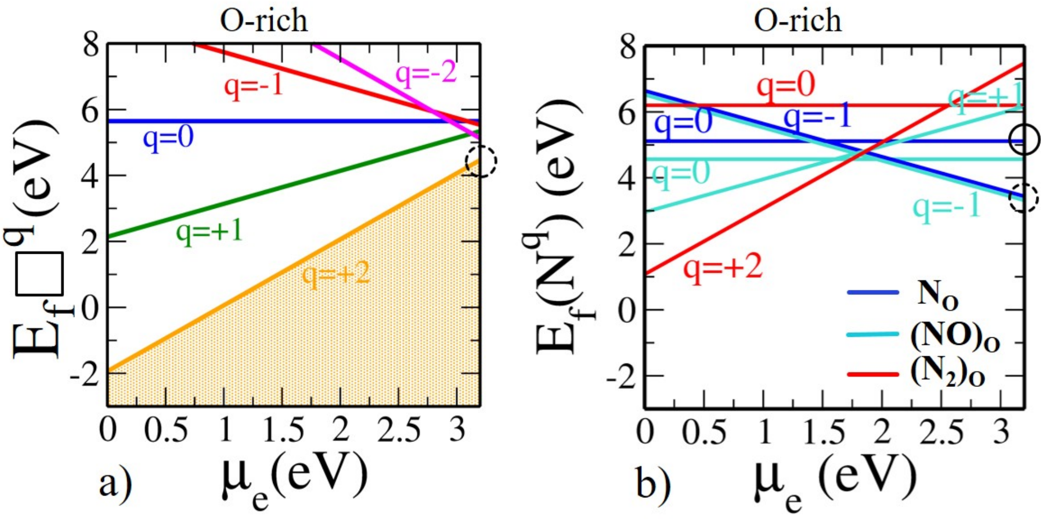

Figure S4: Formation energy plot for a) single O-vacancy b) N-related defects at O-rich condition.

Presence of Oxygen vacancies in undoped and doped TiO_2

It has been widely reported that TiO_2 can be easily reduced, and it supports a high degree of nonstoichiometry in the form of oxygen vacancies. The removal of an oxygen atom from the TiO_2 lattice results in a doubly occupied single-particle state in the band gap, near the conduction-band minimum (CBm). Therefore, two electrons are thus transferred to the CBm, rendering it impossible to stabilize a neutral or +1 charge state of the vacancy. Thus, oxygen vacancy act as a shallow donor for which the +2 charge state is lower in energy than the neutral and +1 charge states for all the values of μ_e (reservoir of electrons varied from VBM to CBm) shown in (Figure S3). Note that, the chemical potential of oxygen $\Delta\mu_O$ varies as - 4.11 eV (O-poor), -1.5 eV (O-intermediate) and 0 eV (O-rich) respectively. We have found that for the case of anatase TiO_2, the formation energy of oxygen vacancy with +2 charge state is relatively low in n-type TiO_2 under O-poor conditions but it rapidly increases with the oxygen chemical potential as shown by dotted circle in (Figure S3). However, in the literature, people have reported that O-vacancies degrade the photocatalytic activity by generating the recombination centre [7, 8]. But photocatalytic activity can be enhanced by considering oxygen vacancies together with non-metal dopants. Note that, oxygen vacancies are not neglected in our case, even for the case of non-metal doping. It is well known fact that O-vacancies are responsible for n-type conductivity in TiO_2 anatase (see reference [9]). The formation energy required to form single O-vacancy in TiO_2 is 4.46 eV near CBm, shown by dotted circle in (Figure S4(a)). Now, if we substitute Nitrogen at the place of oxygen $(N)_O$ with neutral charge, it requires 5.11 eV energy, shown by solid circle in (Figure S4(b)). It means the substitution of nitrogen at the place of oxygen is unfavorable, because its formation energy (5.11 eV) is higher as compare to formation energy of single oxygen vacancy (4.46 eV). The obvious reason for that is charge un-compensation. Now, if we substitute Nitrogen at the oxygen site $(N)_O$ with -1 charge state, the formation energy obtained is 3.44 eV, as shown by dotted circle in (Figure S4(b)) above. This means if we assume of having reservoir of electrons, from where the dopant can trap one electron, the dopant will be stabilized at the defect site. This is as good as claiming the presence of having several oxygen vacancies in the lattice with uncompensated electrons and those electrons can be trapped by the external dopant. Thus, we can adapt either of the approaches i.e. (1) addition of external charge to the dopant or (2) explicit presence of oxygen vacancy for the neutral dopants.

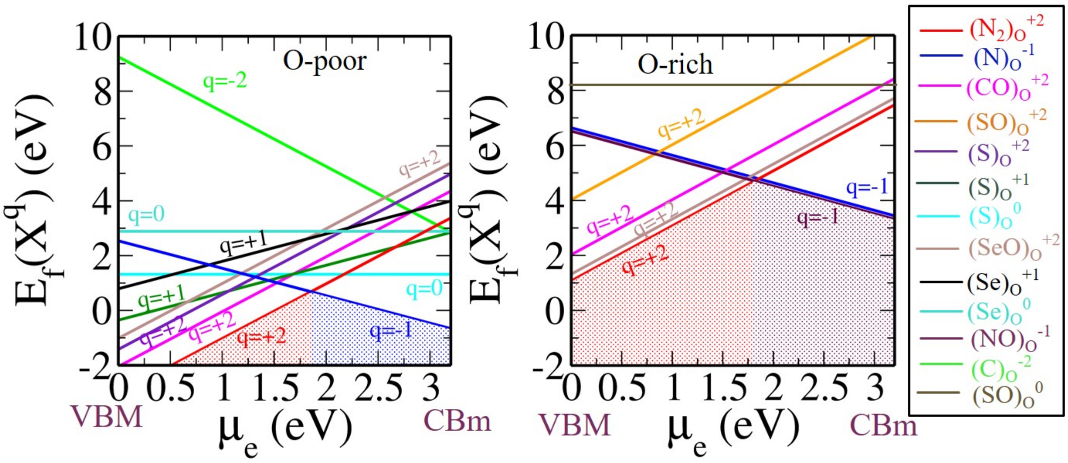

Figure S5: Formation energy for X-related defects that include the most stable defects obtained from 3D phase diagram in anatase $Ti_{16}O_{32}$ at O-poor and O-rich conditions.

Comparison among non-metal dopants (X= N, C, S, Se) at O-poor and O-rich conditions.

It is shown in Figure S5 that at O-poor condition $(N_2)_O^{+2}$ configuration is most stable at the lower value of μ_e (p-type doping) and $(N)_O^{-1}$ is most stable at the higher value of μ_e (n-type doping). And finally at O-rich condition, again the $(N_2)_O^{+2}$ configuration is stable at the lower value of μ_e, whereas $(NO)_O^{-1}$ configuration is stable at the higher value of μ_e.

Formation energy formula for C related defects

The formation energy of $(C)_O$ in charge state q is given below.

$$\mathrm{E_f}[(\mathrm{C})_\mathrm{O}^q] = \mathrm{E}[\mathrm{CTi_{16}O_{31}}]^q - \mathrm{E}[\mathrm{Ti_{16}O_{32}}]^0 + \frac{1}{2}\mathrm{E}[\mathrm{O_2}] + \Delta\mu_\mathrm{O} - \frac{1}{8}\mathrm{E}[\mathrm{C_8}] - \Delta\mu_\mathrm{C} + q(\mu_\mathrm{e} + \mathrm{VBM} + \Delta\mathrm{V}) \tag{3}$$

The formation energy for interstitial C i.e., $(CO)_O$ is given by:

$$\mathrm{E_f}[(\mathrm{CO})_\mathrm{O}^q] = \mathrm{E}[\mathrm{CTi_{16}O_{32}}]^q - \mathrm{E}[\mathrm{Ti_{16}O_{32}}]^0 - \frac{1}{8}\mathrm{E}[\mathrm{C_8}] - \Delta\mu_\mathrm{C} + q(\mu_\mathrm{e} + \mathrm{VBM} + \Delta\mathrm{V}) \tag{4}$$

And, formation energy for C complex in which interstitial C sharing a lattice site with $(C)_O$ i.e., $(C_2)_O$ is given by:

$$\mathrm{E_f}[(\mathrm{C_2})_\mathrm{O}^q] = \mathrm{E}[\mathrm{C_2Ti_{16}O_{31}}]^q - \mathrm{E}[\mathrm{Ti_{16}O_{32}}]^0 + \frac{1}{2}\mathrm{E}[\mathrm{O_2}] + \Delta\mu_\mathrm{O} - 2\Delta\mu_\mathrm{C} - \frac{1}{4}\mathrm{E}[\mathrm{C_8}] + q(\mu_\mathrm{e} + \mathrm{VBM} + \Delta\mathrm{V}) \tag{5}$$

Formation energy formula for S related defects

The formation energy of $(S)_O$ in charge state q is given as:

$$\mathrm{E_f}[(\mathrm{S})_\mathrm{O}^q] = \mathrm{E}[\mathrm{STi_{16}O_{31}}]^q - \mathrm{E}[\mathrm{Ti_{16}O_{32}}]^0 + \frac{1}{2}\mathrm{E}[\mathrm{O_2}] + \Delta\mu_\mathrm{O} - \frac{4}{32}\mathrm{E}[\mathrm{S_8}] - \Delta\mu_\mathrm{S} + q(\mu_\mathrm{e} + \mathrm{VBM} + \Delta\mathrm{V}) \tag{6}$$

The formation energy for interstitial S i.e., $(SO)_O$ is given by:

$$\mathrm{E_f}[(\mathrm{SO})_\mathrm{O}^q] = \mathrm{E}[\mathrm{STi_{16}O_{32}}]^q - \mathrm{E}[\mathrm{Ti_{16}O_{32}}]^0 - \frac{4}{32}\mathrm{E}[\mathrm{S_8}] - \Delta\mu_\mathrm{S} + q(\mu_\mathrm{e} + \mathrm{VBM} + \Delta\mathrm{V}) \tag{7}$$

Similarly, formation energy for S complex in which interstitial S sharing a lattice site with $(S)_O$ i.e., $(S_2)_O$ is given by:

$$\mathrm{E_f}[(\mathrm{S_2})_\mathrm{O}^q] = \mathrm{E}[\mathrm{S_2Ti_{16}O_{31}}]^q - \mathrm{E}[\mathrm{Ti_{16}O_{32}}]^0 + \frac{1}{2}\mathrm{E}[\mathrm{O_2}] + \Delta\mu_\mathrm{O} - 2\Delta\mu_\mathrm{S} - \frac{4}{16}\mathrm{E}[\mathrm{S_8}] + q(\mu_\mathrm{e} + \mathrm{VBM} + \Delta\mathrm{V}) \tag{8}$$

Formation energy formula for Se related defects

The formation energy of $(\mathrm{Se})_\mathrm{O}$ in charge state q is therefore given by:

$$\mathrm{E_f}[(\mathrm{Se})_\mathrm{O}^q] = \mathrm{E}[\mathrm{SeTi_{16}O_{31}}]^q - \mathrm{E}[\mathrm{Ti_{16}O_{32}}]^0 + \frac{1}{2}\mathrm{E}[\mathrm{O_2}] + \Delta\mu_\mathrm{O} - \mathrm{E}[\mathrm{Se}] - \Delta\mu_\mathrm{Se} + q(\mu_\mathrm{e} + \mathrm{VBM} + \Delta\mathrm{V}) \tag{9}$$

The formation energy for interstitial Se i.e., $(\mathrm{SeO})_\mathrm{O}$ is given by:

$$\mathrm{E_f}[(\mathrm{SeO})_\mathrm{O}^q] = \mathrm{E}[\mathrm{SeTi_{16}O_{32}}]^q - \mathrm{E}[\mathrm{Ti_{16}O_{32}}]^0 - \mathrm{E}[\mathrm{Se}] - \Delta\mu_\mathrm{Se} + q(\mu_\mathrm{e} + \mathrm{VBM} + \Delta\mathrm{V}) \tag{10}$$

And, formation energy for Se complex in which interstitial Se sharing a lattice site with $(\mathrm{Se})_\mathrm{O}$ i.e., $(\mathrm{Se_2})_\mathrm{O}$ is given by:

$$\mathrm{E_f}[(\mathrm{Se_2})_\mathrm{O}^q] = \mathrm{E}[\mathrm{Se_2Ti_{16}O_{31}}]^q - \mathrm{E}[\mathrm{Ti_{16}O_{32}}]^0 + \frac{1}{2}\mathrm{E}[\mathrm{O_2}] + \Delta\mu_\mathrm{O} - 2\Delta\mu_\mathrm{Se} - 2\mathrm{E}[\mathrm{Se}] + q(\mu_\mathrm{e} + \mathrm{VBM} + \Delta\mathrm{V}) \tag{11}$$

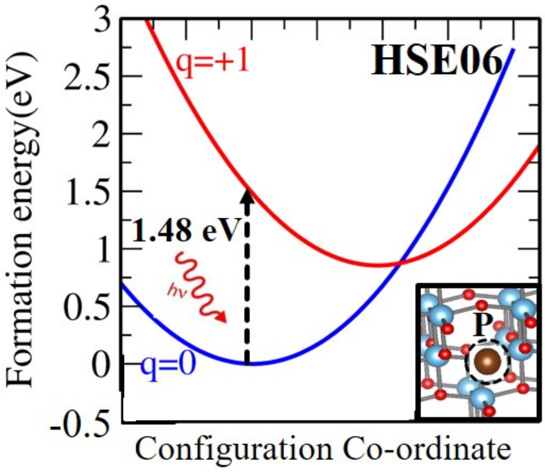

Figure S6: Configuration Co-ordinate diagram for Phosphorous doped TiO_2.

We have initiated with non-metal dopants X (=Nitrogen (N), Carbon (C), Sulphur (S), Selenium (Se), Phosphorous (P) etc.) to substitute at oxygen site in TiO_2 anatase to estimate which non-metal dopants are suitable to have a response in the visible region. We have found that, only these non-metal dopants (viz. N, C, S and Se) have absorption peak lying in the visible region range (1.55 - 3.0 eV) (shown in the validation part Figure 2 in main manuscript), while the others fall in IR region. Indeed, for the case of P doping, we have found reduction in the bandgap (similar to other dopants) but here, we are more interested on the dopants that have a response particularly, in the visible region range. This point is clear from our simulated configuration coordinate diagram, (Figure S6) which shows the response of P doping in the infrared region (1.48 eV). Therefore, we have not considered P doping for further study.

References

[1] Bhattacharya, S., Berger, D., Reuter, K., Ghiringhelli, L. M. & Levchenko, S. V. Theoretical evidence for unexpected O-rich phases at corners of MgO surfaces. *Phys. Rev. Mater.* **1**, 071601 (2017).

[2] Heyd, J., Scuseria, G. E.& Ernzerhof, M. Hybrid functionals based on a screened coulomb potential. *The J. Chem. Phys.* **118**, 8207–8215 (2003).

[3] Krukau, A. V., Scuseria, G. E., Perdew, J. P. & Savin, A. Hybrid functionals with local range separation. *The J. Chem. Phys.* **129**, 124103 (2008).

[4] Deák, P., Aradi, B., Frauenheim, T., Janzén, E. & Gali, A. Accurate defect levels obtained from the HSE06 range-separated hybrid functional. *Phys. Rev. B* **81**, 153203 (2010).

[5] Bhattacharya, S., Sonin, B. H., Jumonville, C. J., Ghiringhelli, L. M. & Marom, N. Computational design of nanoclusters by property-based genetic algorithms: Tuning the electronic properties of $(TiO_2)_n$ clusters. *Phys. Rev. B* **91**, 241115 (2015).

[6] Bhattacharya, S., Levchenko, S. V., Ghiringhelli, L. M. & Scheffler, M. Efficient ab initio schemes for finding thermodynamically stable and metastable atomic structures: Benchmark of cascade genetic algorithms. *New J. Phys.* **16**, 123016 (2014).

[7] Xie, T.-H., & Lin, J. Origin of photocatalytic deactivation of TiO_2 film coated on ceramic substrate. *J. Phys. Chem. C* **111**, 9968 (2007).

[8] Zhang, Z., et al. Band-gap tuning of n-doped TiO_2 photocatalysts for visible-light-driven selective oxidation of alcohols to aldehydes in water. *Rsc Advances* **3**, 7215 (2013)

[9] Janotti, A., Varley, P., Umezawa, N., Kresse G. & Van de Walle, C. Hybrid functional studies of the oxygen vacancy in TiO_2. *Phys. Rev. B* **81**, 085212 (2010).
